# Effect of subjective vertical perception on lateral flexion posture of patients with Parkinson’s disease

**DOI:** 10.1038/s41598-022-05587-z

**Published:** 2022-01-27

**Authors:** Kyohei Mikami, Makoto Shiraishi, Tsutomu Kamo

**Affiliations:** 1 Department of Rehabilitation, Noborito Neurology Clinic, Kawasaki, Japan; 2 Department of Neurology, St. Marianna University School of Medicine, Kawasaki, Japan; 3 Department of Neurology, Noborito Neurology Clinic, Kawasaki, Japan

**Keywords:** Diseases, Medical research, Neurology, Signs and symptoms

## Abstract

In a retrospective study we tested our hypothesis that the subjective postural vertical ratio (SPV ratio), i.e., the subjective postural vertical measured in relation to the lateral flexion axis, is predictive of lateral trunk flexion in patients with Parkinson's disease (PD). Twenty-five patients were included. The SPV angle, i.e., the subjective perception of a vertical position with reference to the vertical axis, and the SPV ratio, i.e., the SPV angle with reference to the axis of lateral flexion, were calculated. The SPV ratio (r = 0.698, *P* = 0.001) and LTF angle (r =  − 0.601, *P* = 0.001) correlated with change in the LTF angle at 1 year. The SPV ratio was significantly smaller in patients for whom lateral trunk flexion improved (n = 12) than in those for whom it did not improve (n = 13) (0.99 ± 0.78 vs 1.66 ± 0.71, *P* = 0.011). The AUC under the ROC curve of the SPV ratio for discrimination of improvement was 0.795 (95% confidence interval: 0.61–0.98). We found that the SPV ratio is associated with change in the LTF and that it can conceivably be used to predict the likelihood of improvement in PD-associated lateral trunk flexion.

## Introduction

Lateral trunk flexion, a postural abnormality characterized by lateral deviation of the spine and the corresponding tendency to lean to one side, is common in patients with Parkinson’s disease (PD), with its prevalence, including that of mild lateral flexion, reported to exceed 40% among patients with PD of Hoehn and Yahr stages 1–4 and to exceed 80% among patients with PD of stage 4^1^. When first recognized in patients with PD, the lateral trunk flexion is typically mild or moderate^2^. Lateral trunk flexion affects patients’ balance^3^, contributes to low back pain^2^ and correlates with health-related quality of life^4,5^. The lateral trunk flexion itself requires treatment because it and other postural abnormalities associated with PD are not responsive to antiparkinson drugs typically administered to patients with PD^6^.

Postural abnormalities associated with PD are complex and result from various factors^7^. One contributing factor is patients’ subjective postural vertical^8^, which refers to patients’ tendency to incorrectly perceive themselves to be standing upright when they are in fact leaning to one side. We showed previously that for patients who exhibit lateral trunk flexion, their subjective postural vertical is shifted in the lateral bending direction^9^, and we also showed that patients’ baseline subjective postural vertical affects their forward bending posture assessed 1 year later^10^.

Rehabilitation therapy that focuses on postural awareness and active self-correction^11^ has shown success as treatment for PD-associated lateral trunk flexion, and rehabilitation based on proprioceptive and tactile stimulation^12^ has also shown success. However, improvement is achieved only in some cases, with a poor response attributed in part to lack of translation of the therapy content into daily life. We hypothesized that lateral inclination of the subjective postural vertical influences the course of lateral flexion in patients with PD, even in those who have undergone rehabilitation therapy. To test our hypothesis, we conducted a retrospective study in which we investigated the effect of the lateral subjective vertical on the course of lateral trunk flexion of patients who underwent rehabilitation therapy at regular intervals.

## Results

### Patient characteristics

The study group comprised 25 patients (13 men and 12 women) with PD-associated lateral trunk flexion and who were evaluated regularly for at least 1 year, with variables assessed at the first evaluation considered baseline variables. As shown in Table 1, patients’ baseline age was 72.9 ± 8.3 (range 56–93) years, disease duration was 4.9 ± 4.8 (range 0–20) years, modified Hoehn and Yahr stage was 2.7 ± 0.5 (range 2–4), Unified Parkinson's Disease Rating Scale part III score was 13.9 ± 7.8 (range 2–30), lateral trunk flexion angle (LTF angle) was 5.6 ± 6.4° (range 1–26°), subjective postural vertical angle (SPV angle) was 4.6 ± 3.5° (range 1.5–17.8°) and subjective postural vertical ratio (SPV ratio) was 1.4 ± 0.8 (range 0.2–3.3). At 1 year, the LTF angle had decreased by 1° or more in 12 patients (improved LTF group) but not in the other 13 patients (non-improved LTF group).

**Table 1:** Characteristics of the study patients at the time of initial evaluation (n = 25).

Age (years)	72.9 ± 8.3 (72: 56–93)
Sex ratio (M/F)	13/12
Disease duration (years)	4.9 ± 4.8 (3:0–20)
mH&Y score	2.7 ± 0.5 (3:2–4)
UPDRS I score	1.4 ± 1.6 (1:0–6)
UPDRS II score	6.8 ± 3.9 (8: 0–13)
UPDRS III score	13.9 ± 7.8 (14: 2–30)
UPDRS IV score	1.8 ± 2.4 (1:0–8)
UPDRS total score	24.0 ± 12.0 (24:5–46)
MMSE score	27.8 ± 2.2 (28:23–30)
MoCa-J score	23.4 ± 3.5 (24:15–29)
FAB score	14.9 ± 1.9 (15:11–18)
FTF (°)	12.7 ± 8.0 (12:2–33)
LTF (°)	5.6 ± 6.4 (4:1–26)
SPV angle (°)	4.6 ± 3.5 (3.3:1.5–17.8)
SPV ratio	1.4 ± 0.8 (1.2:0.2–3.3)
LEDD (mg)	598.4 ± 320.3 (530:150–1514.5)
LEDD_DA_ (mg)	213.8 ± 255.7 (150:0–865.5)

Mean ± SD (median: range) values are shown, unless otherwise indicated.

*mH&Y* modified Hoehn and Yahr stage, *UPDRS* Unified Parkinson’s Disease Rating Scale, *MMSE* Mini-Mental State Examination, *MoCa-J* Japanese version of Montreal Cognitive Assessment, *FAB* Frontal Assessment Battery, *FTF* forward trunk flexion, *LTF* lateral trunk flexion, *SPV* subjective postural vertical, *LEDD* levodopa equivalent daily dose, *LEDD*_*DA*_ levodopa equivalent daily dose of dopamine agonist.

### Relation between LTF and change in the LTF angle

**Figure 1:**
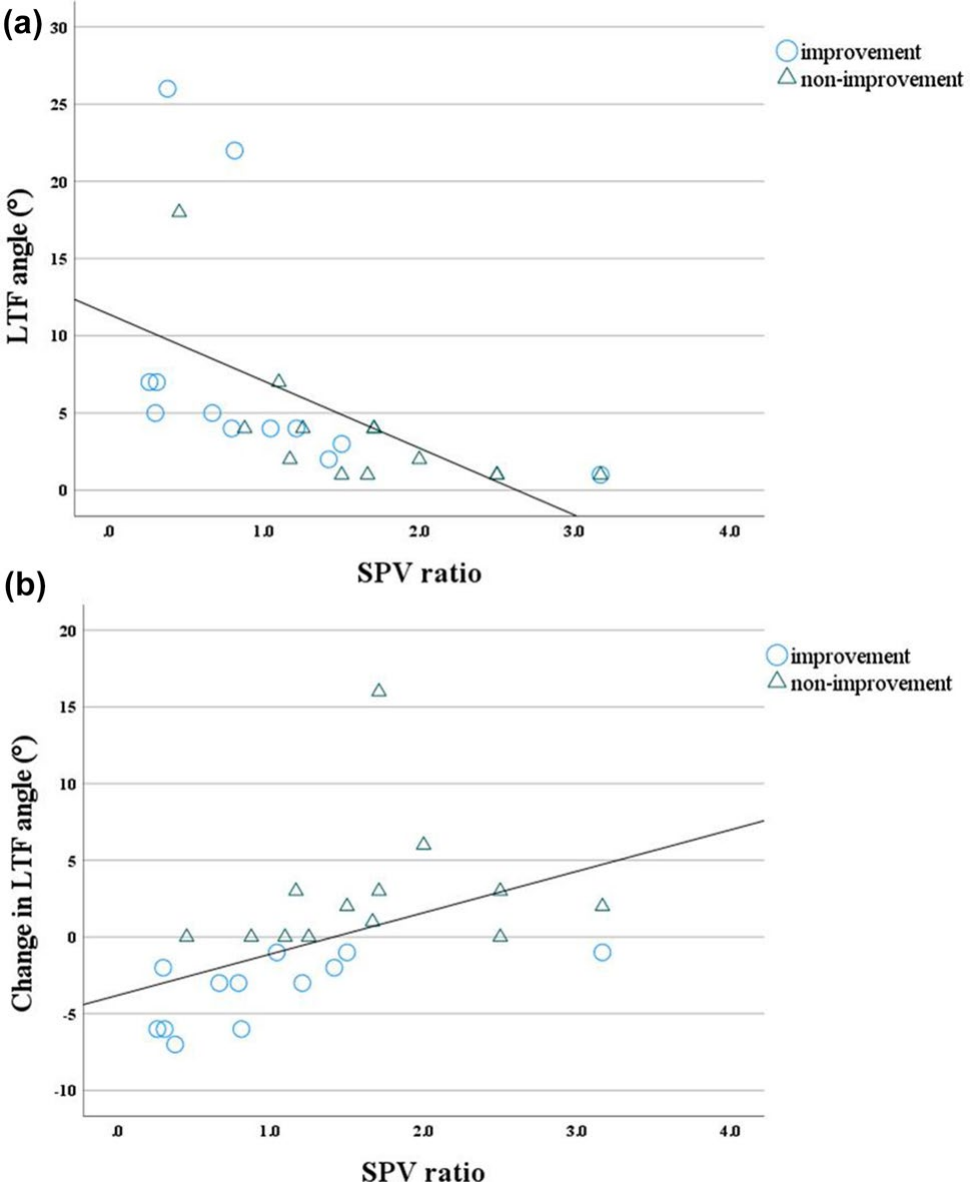
Scatter plots showing relations between (**a**) baseline LTF angle and baseline SPV ratio and (**b**) change in the LTF angle at 1 year and baseline SPV ratio. SPV subjective postural vertical, LTF lateral trunk flexion.

Negative correlation was found between patients’ baseline LTF angle and baseline SPV ratio (r =  − 0.838, *P* = 0.001) (Fig. 1a), whereas positive correlation was found between patients’ baseline LTF angle and baseline SPV angle (r = 0.506, *P* = 0.010). Positive correlation was found between change in the LTF angle observed at 1 year (calculated as the difference between the baseline LTF angle and the LTF angle determined at 1 year) and the baseline SPV ratio (r = 0.698, *P* = 0.001 (Fig. 1b), and negative correlation was found between change in the LTF angle and the baseline LTF angle (r =  − 0.601, *P* = 0.001). No relation was observed between change in the LTF angle and age, disease duration, SPV angle, forward trunk flexion angle (FTF angle), modified Hahn and Yahr stage, Unified Parkinson's Disease Rating Scale-part III score, total Unified Parkinson's Disease Rating Scale score, levodopa equivalent daily dose or levodopa equivalent daily dose of dopamine agonist.

### Results of comparison of patient characteristics

**Figure 2:**
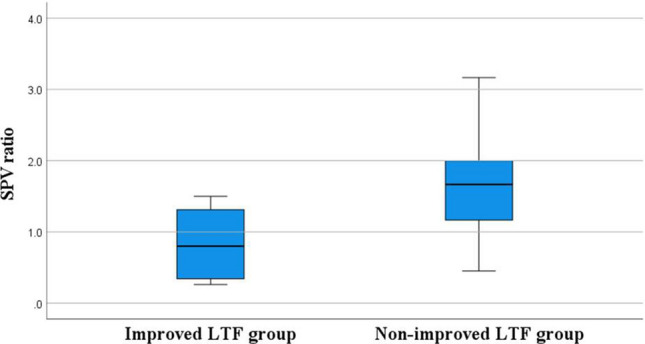
Box and whisker plot showing distribution of the SPV ratios in the improved LTF group and non-improved LTF group.

We investigated whether there was a differences in the SPV ratio between the improved LTF group and non-improved LTF group. We found the SPV ratio to be significantly smaller in the improved LTF group than in the non-improved LTF group (0.99 ± 0.78 vs. 1.66 ± 0.71, *P* = 0.011) (Fig. 2).

We also investigated whether there was any difference between the improved LTF group and non-improved LTF group in patient characteristics. A significant difference was found in the UPDRS IV score (Table 2).

**Table 2:** Characteristics of the study patients, per group.

	Improved LTF group (n = 12)	Non-improved LTF group (n = 13)	*P* value
Age (years)	72.0 ± 8.3 (73.5: 56–84)	75.6 ± 7.8 (72: 64–93)	0.73
Sex ratio (M/F)	5/7	8/5	0.32
Disease duration (years)	4.6 ± 5.3 (2: 1–19)	5.2 ± 4.3 (5: 0–15)	0.65
mH&Y score	2.9 ± 0.5 (3: 2–4)	2.6 ± 0.4 (2.5: 2–3)	0.14
UPDRS I score	1.3 ± 1.5 (0.5: 0–5)	1.6 ± 1.6 (1: 0–6)	0.54
UPDRS II score	5.3 ± 4.2 (6: 0–12)	8.2 ± 2.9 (8: 3–13)	0.11
UPDRS III score	15.1 ± 7.6 (15: 5–30)	12.8 ± 7.8 (13: 2–26)	0.41
UPDRS IV score	1.0 ± 2.2 (0: 0–8)	2.5 ± 2.3 (1: 0–7)	**0.03**
UPDRS total score	22.7 ± 12.1 (22: 5–43)	25.2 ± 11.7 (24: 7–46)	0.57
MMSE score	28.0 ± 2.2 (28.5: 23–30)	27.6 ± 2.2 (27: 24–30)	0.69
MoCa-J score	22.8 ± 3.4 (23.5: 16–28)	24.0 ± 3.5 (24: 15–29)	0.47
FAB score	15.2 ± 2.1 (15: 11–18)	14.7 ± 1.6 (15: 12–17)	0.50
FTF (°)	12.2 ± 8.5 (10.5: 2–33)	13.2 ± 7.4 (12: 4–30)	0.69
LTF (°)	7.5 ± 7.6 (4.5: 1–26)	3.8 ± 4.5 (2: 1–18)	0.05
SPV angle (°)	4.9 ± 4.4 (3.3: 1.5–17.8)	4.3 ± 2.3 (3.5: 1.5–8.2)	0.94
LEDD (mg)	523.9 ± 262.7 (437.5: 150–1031.8)	667.2 ± 351.8 (560.0: 300–1515.5)	0.32
LEDD_DA_ (mg)	223.3 ± 220.0 (211.4: 0–618.8)	205.1 ± 284.4 (150: 0–865.5)	0.57

Significant values in bold.

Mean ± SD (median: range) values are shown, unless otherwise indicated.

*mH&Y* modified Hoehn and Yahr stage, *UPDRS* Unified Parkinson’s Disease Rating Scale, *MMSE* Mini-Mental State Examination, *MoCa-J* Japanese version of Montreal Cognitive Assessment, *FAB* Frontal Assessment Battery, *FTF* forward trunk flexion, *LTF* lateral trunk flexion, *SPV* subjective postural vertical, *LEDD* levodopa equivalent daily dose, *LEDD*_*DA*_ levodopa equivalent daily dose of dopamine agonist.

When we compared baseline daily doses of PD medications and daily doses of the medications 1 year later in the improved LTF group, we found no significant difference in the levodopa equivalent daily dose (620.6 mg ± 220.9 vs. 463.1 mg ± 172.0) or the levodopa equivalent daily dose of dopamine agonist (209.6 mg ± 210.5 vs. 97.3 mg ± 116.6). Comparison in the non-improved LTF group also revealed no significant difference in the levodopa equivalent daily dose (577.9 ± 389.1 vs. 670.8 ± 269.9) or the levodopa equivalent daily dose of dopamine agonist (217.7 ± 291.2 vs. 139.1 ± 157.8).

### Receiver-operating characteristic (ROC) curve analysis

**Figure 3:**
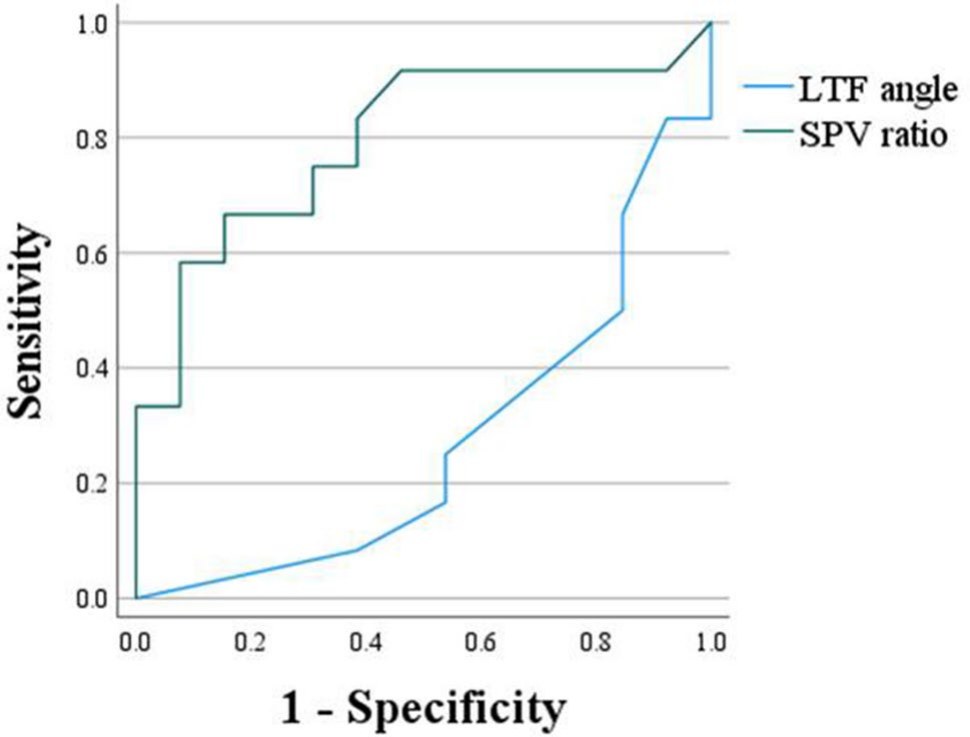
ROC curves showing performance of the SPV ratio and LTF angle in the improved LTF group.

An ROC curve was created to investigate utility of the LTF angle and of the SPV ratio, both of which were found to correlate with change in the LTF angle at 1 year, for predicting change in the LTF angle at 1 year. The two AUCs were compared. The AUC for the SPV ratio in the improved LTF group was 0.795 (95% confidence interval [CI] 0.61–0.98), and that for the LTF angle was 0.272 (95% CI 0.07 − 0.47), with the AUC for the SPV ratio being significantly larger (*P* = 0.006, 95% CI − 0.90 to − 0.150). The cut-off value for the SPV ratio in the improved LTF group was 1.07, with a sensitivity of 67% and specificity of 85% (Fig. 3).

## Discussion

This study of patients with PD-associated lateral trunk flexion yielded two main findings. The first was positive correlation between patients’ baseline SPV ratio, i.e., the subjective postural vertical with reference to the axis of lateral flexion at the time of initial evaluation, and the change in patients’ LTF angle 1 year later. The second was the significantly lower baseline SPV ratio in the improved LTF group than in the non-improved LTF group, with a cut-off value of 1.07 being predictive of improvement in the LTF angle.

Although positive correlation was observed between the SPV ratio and the change in LTF at 1 year, no correlation was found between the SPV angle and the change in LTF at 1 year. The SPV ratio is used to evaluate the subjective postural vertical with reference to the axis of lateral flexion, so an SPV ratio of ≥ 1 means that the SPV angle is larger than the LTF angle. In a previously reported cross-sectional study, cases in which the SPV angle was larger than the LTF angle were observed, suggesting use of the SPV ratio as a predictor of the course of lateral trunk flexion^9^. Our study, reported herein, verified a relation between the SPV ratio and change in the LTF angle at 1 year, confirming potential usefulness of the SPV ratio obtained on initial evaluation as a prognosticator of the course of PD-associated lateral trunk flexion.

Various sensory inputs are involved in vertical perception, including visual input, vestibular input and somatic input^13^, but when we measured the subjective postural vertical, our study patients were required to close their eyes, which eliminated visual input. In healthy individuals, somatic input is reported to have a greater effect on the subjective postural vertical than vestibular sensation^14^, and the subjective postural vertical values in patients with PD have been shown to be greater than those in age-matched healthy individuals^8^. In other words, the subjective postural vertical, which is perceived primarily somatically, tends to increase in patients with PD. In addition, the subjective postural vertical begins to increase during the early stages of PD, indicating the necessity of evaluating the subjective postural vertical in patients with early PD. Results of our study suggest that the initially obtained SPV ratio can be used to predict long-term changes in lateral trunk flexion. As to the SPV ratio, UPDRS IV was also found to be a factor related to the course of lateral flexion (Table 2). This result shows that motor complications are often seen in patients with poor control of lateral flexion posture. It was speculated that in cases where the therapeutic effect was not stable, poor recognition of the vertical position made the patient resistant to rehabilitation. Clinical background factors for postural deterioration in PD patients include disease duration, severity, antiparkinsonian drugs^7^ and cognitive function^15^. Our study patients’ Montreal Cognitive Assessment (Japanese version) scores were lower than the cut-off value for mild cognitive impairment^16^. As cognitive function is known to be related to postural dynamics in patients with PD^15^, further investigation is needed regarding the involvement of cognitive decline in postural deterioration.

We found no correlation between change in the LTF angle at 1 year and the levodopa equivalent daily dose or levodopa equivalent daily dose of dopamine agonist. We also found no significant difference between the baseline levodopa equivalent daily dose or the levodopa equivalent daily dose of dopamine agonist and amounts administered at 1 year in the improved LTF group or in the non-improved LTF group. Some investigators have indicated that switching or reducing anti-PD drugs improves lateral trunk flexion^17,18^, whereas others have indicated that most patients with PD-related lateral trunk flexion do not experience improvement in the lateral trunk flexion after switching anti-PD drugs^6^, so the effects of these drugs on lateral trunk flexion remain unclear. When evaluating patients for inclusion in the present study, we excluded patients who had switched their oral medication within 1 week prior to evaluation, and we also excluded patients who presented with postural abnormalities that had appeared within 1 month prior to evaluation, so we believe that oral medications had very little effect on the improvement in lateral trunk flexion that we observed in some patients.

Our findings should be interpreted in light of our study limitations. First, all patients included were from a single institution, increasing the likelihood of selection bias and information bias. Second, posture-related study variables were assessed in the coronal plane, but lateral trunk flexion in patients with PD is frequently complicated by forward trunk flexion^5^. It seems that, in addition to the SPV ratio and SPV angle, a three-dimensional index should be incorporated into the evaluation. However, for three-dimensional analysis, a large apparatus, such as Spacecurl, is required^19^, and the challenge is to develop a simple evaluation method that is easy to use in daily clinical practice. Third, this study clarified the fact that the SPV ratio is useful for predicting improvement in the LTF angle at 1 year but not improvement in the subjective postural vertical itself. We are aware that background clinical factors are related to postural deterioration in patients with PD. As noted above, such factors include disease duration, disease severity, non-use of antiparkinsonian drugs, and cognitive impairment^15,16^. Our patients’ mean Montreal Cognitive Assessment (Japanese version) score was 23, with a value of < 26 indicative of cognitive impairment^16^. Our patients’ scores point to a need for future investigation into the nature of the relation between patients’ cognitive status and postural deterioration. At present, we are focused simply on the relation between patients’ subjective postural vertical and postural deterioration, lateral trunk flexion in particular.

In summary, our study showed that a patient’s initially determined subjective postural vertical with reference to the axis of lateral flexion (i.e., the SPV ratio) influences lateral trunk flexion evaluated by the time 1 year has passed. Thus, the SPV ratio shows promise for use as a predictor of improvement in lateral trunk flexion in patients with PD.

## Methods

### Patient selection

Patients included in the study were selected from among adult patients with PD who had visited the outpatient rehabilitation department of Noborito Neurology Clinic regularly for at least 1 year, starting sometime between March 2018 and March 2019. All patients included had lateral trunk flexion for which there had been no surgical intervention. Rather, they had undergone rehabilitation therapy for the lateral trunk flexion. The LTF angle had been measured on initial examination and at 1 year following the initial examination in all patients included in the study. Patients chosen for inclusion met the study criteria; i.e., their PD had been clinically established according to the Movement Disorder Society clinical diagnostic criteria for Parkinson’s disease^20^; they had been judged able to complete a 1-year evaluation; they were of age 20 years or more; there had been no change in oral medications within 1 week of the initial or 1-year evaluation; they were in an “on” state at the time of initial or 1-year evaluation if suffering from the “wearing-off” phenomenon; they had been judged able to understand instructions; and they were able to stand upright. Exclusion criteria were as follows: diagnosis of a PD-related disorder other than PD itself; presences of any psychiatric symptoms, such as visual or other hallucinations at the time of initial or 1-year evaluation; signs of the wearing-off phenomenon during the drug administration period. Wearing off was determined using UPDRS IV and the symptom diary; restricted range of motion that rendered passive guidance of the patient into a vertical position impossible; dramatic worsening of the PD symptoms within 1 week prior to evaluation; and rapid onset of postural abnormalities, i.e., onset within 1 month prior to evaluation.

Informed consent was obtained from all patients and/or their family. Patients had been informed that they could decide not to approve use of their clinical data for study purposes and had been given directions on how to “opt out” from the use of their data for such purposes. They were told that they would incur no disadvantages if they did not consent to the use of their data. Furthermore, patients’ data were anonymized to ensure that no personal identifying information was disclosed. The study was conducted in accordance with the study guidelines and regulations of St. Marianna University School of Medicine and with the approval of the St. Marianna University School of Medicine ethics committee (approval number: 5004).

### Rehabilitation program

All study patients had undergone 60 min of rehabilitation therapy once a week or more between the time of the initial assessment and the time of final assessment 1 year later. The rehabilitation program was designed with three objectives in mind^11,21,22^. The first was to appropriately correct the patient’s posture, the second was to enable the patient to perform various physical exercises, including maintaining control of the corrected trunk, and the third was to increase the patient’s endurance. Each session was of three separate components: a 20-min period that entailed the patient focusing on somatic sensations while the physical therapist made adjustments to correct for the difference between the patient’s actual posture and the vertical axis, a 20-min period designed to help the patient maintain control of the trunk while adopting various positions and performing various exercises, and a 10-min period of aerobic exercise performed on an ergometer or treadmill.

### Postural evaluation

As noted above, patients’ lateral trunk flexion had been evaluated at the initial examination and then at 1 year. Patients’ subjective postural vertical had been evaluated at the initial examination. Standing posture at rest and the subjective postural vertical were taken as the postural endpoints. In accordance with preceding studies^9^, the landmark points for postural evaluation were the spinous processes of the seventh cervical (C7) and fifth lumbar (L5) vertebrae, and reflective markers for three-dimensional motion analysis (Nobbyteck VNS-BL-MC-190) were attached to these sites. Image analysis software Image J (https://imagej.nih.gov/ij/index.html) was used to measure the relevant angles. Images were captured at rest using a digital camera (Panasonic DMC-LZ10), after which it was possible to use the software to calculate the angle between the vertical axis and an arbitrary second axis.

### Standing posture at rest

Patients’ LTF and FTF angles had been measured at the time of initial evaluation and at 1 year. The patient’s position on opening his/her eyes immediately after standing up was evaluated three times, and the mean value was taken as the patient’s standing position. A digital camera was aimed at the center point of Jacoby’s line and at the high point of each iliac crest for measurement of the LTF angle and at a point in the sagittal plane for measurement of the FTF angle. Photographs were taken at rest and included both C7 and L5. The standard axis was taken as a vertical line crossing L5 and descending to the floor. The axis of forward flexion was defined as the line connecting C7 and L5 observed along the sagittal plane, whereas the axis of lateral flexion was defined as a line connecting C7 and L5 observed along the coronal plane^4^.

### Subjective postural vertical evaluation

**Figure 4:**
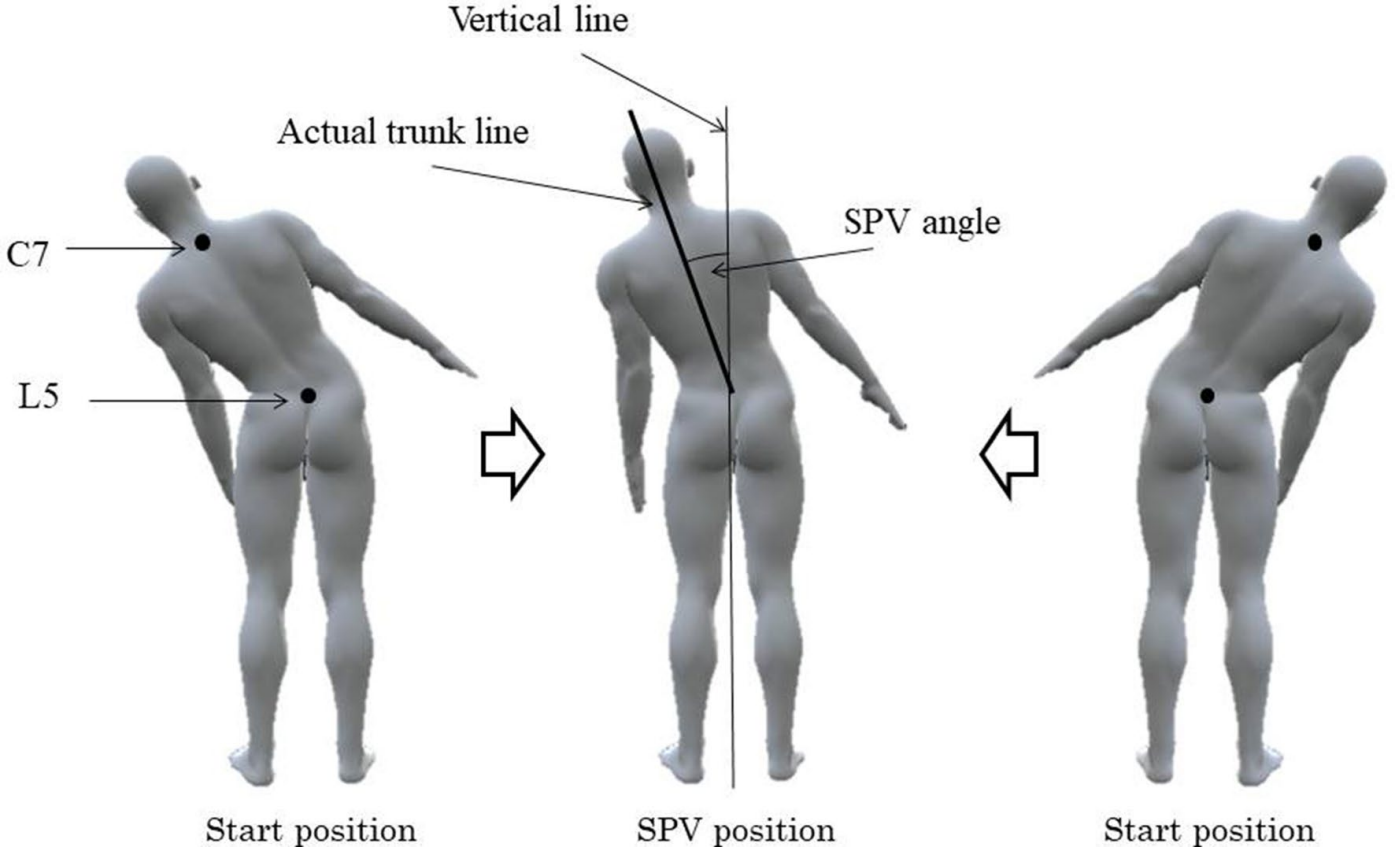
Evaluation of the SPV angle. SPV subjective postural vertical, C7 seventh cervical vertebra, L5 fifth lumbar vertebra.

Patients’ subjective postural vertical was evaluated in the coronal plane, and the SPV angle and SPV ratio were recorded. The SPV angle was defined as the angle between the vertical axis and the axis of lateral flexion. As described previously, the vertical axis was defined as the vertical line passing through L5 and descending to the floor, and was used as the reference, while the axis of lateral flexion was defined as the line connecting C7 and L5 at the point in time when the patient perceived himself/herself to be in a vertical position (Fig. 4)^23^. The SPV angle was measured by first establishing the starting position, which entailed the patient closing their eyes in a standing position and then being passively guided into maximum lateral trunk flexion. The investigator then guided the patient towards the vertical axis. The patient then notified the investigator when they perceived their trunk to be a vertical position, and the images at rest were taken in this position at the height of the Jacoby’s line and along the posterior midline. This sequence was performed three times each on the left and right, giving a total of six measurements. The mean value of these measurements was used as the SPV angle. The SPV ratio was the subjective postural vertical measured with reference to the axis of lateral flexion, and in accordance with preceding studies, was calculated by dividing the SPV angle by the LTF angle (SPV ratio = SPV angle/LTF angle)^9^.

### Data collection and statistical analyses

For the purpose of the study, patients’ medical records were accessed and the following information was obtained: age, sex, disease duration, PD severity and outcomes of neuropsychological examinations. PD severity had been evaluated on the basis of the modified Hoehn and Yahr scale^24^ and the Unified Parkinson’s Disease Rating Scale^25^. The Mini-Mental State Examination^26^ and Japanese version of the Montreal Cognitive Assessment^27^, as well as the Frontal Assessment Battery^28^, were used for neuropsychological testing. Patients’ levodopa equivalent daily dose and levodopa equivalent daily dose of dopamine agonist were also obtained^29^.

Relations between the baseline LTF angle, change in the LTF angle at 1 year and each of the postural evaluation endpoints were analyzed by means of Spearman's rank correlation coefficient. Change in the LTF angle was used to divide the patients into two groups: those for whom LTF improved (LTF improved group) and those for whom LTF did not improve (LTF non-improved group), based on whether or not improvement was observed once or more from the time of initial evaluation to 1 year. Normality of the SPV ratio data was investigated by means of the Shapiro–Wilk Test, and the data were shown to be not normally distributed. Each clinical endpoint was compared using the Mann–Whitney U Test between the improved LTF group and non-improved LTF group. Improvement was defined as a decrease in the LTF angle of 1° or more. EZR^30^ was used for sample size calculation. By using a 4° difference in mean values between the improved LTF group and non-improved LTF group, a standard deviation of 5 for each of the two groups, and an α error level of 0.05, we found the number of cases needed to reach a power level of 0.8 to be 25. To determine the power of the SPV ratio and of the baseline LTF angle for prediction of improvement in patients’ lateral trunk flexion, receiver operating characteristic (ROC) curves were plotted, and areas under the curve (AUCs) were determined. Cut off values were derived on the basis of Youden’s Index (Youden's J statistic)^31^. All statistical analyses were performed with SPSS version 27 (IBM SPSS Statistics for Windows; IBM Corp, Armonk, NY), and *P* < 0.05 was considered significant.
